# Quality of life in Brazilian obese adolescents: effects of a long-term multidisciplinary lifestyle therapy

**DOI:** 10.1186/1477-7525-7-61

**Published:** 2009-07-03

**Authors:** Mara Cristina Lofrano-Prado, Hanna Karen Moreira Antunes, Wagner Luiz do Prado, Aline de Piano, Danielle Arisa Caranti, Lian Tock, June Carnier, Sergio Tufik, Marco Túlio de Mello, Ana R Dâmaso

**Affiliations:** 1 Post-Graduate Program in Nutrition, Federal University of São Paulo/Paulista School of Medicine, Marselhesa Street, 535, São Paulo/SP, Brazil; 2 Department of Health Sciences, Federal University of São Paulo, Baixada Santista Campus, Santos/SP, Brazil; 3 Department of Physical Education, Pernambuco University, Arnóbio Marques Street, 310, Recife/PE, Brazil; 4 Department of Psychobiology, Federal University of São Paulo/Paulista School of Medicine – Marselhesa Street, 500, São Paulo/SP, Brazil

## Abstract

**Background:**

Obesity has adverse physical, social, and economic consequences that can negatively affect quality of life (QOL). Thus the aim of this study was to verify the effects of a long-term multidisciplinary lifestyle intervention on QOL, body image, anxiety, depression and binge eating in obese adolescents.

**Methods:**

Sixty-six obese adolescents (41 girls and 25 boys; BMI: 35.62 ± 4.18 kg/m^2^) were recruited from the Multidisciplinary Obesity Intervention Program outpatient clinic, and were submitted to a multidisciplinary lifestyle therapy (short-term = 12 weeks and long-term = 24 weeks), composed of medical, dietary, exercise and psychological programs. Validated self-report questionnaires were used to assess symptoms of anxiety Trait/State (STAI); depression (BDI); binge eating (BES), body image dissatisfaction (BSQ) and QOL (SF-36). Data were analyzed by means of scores; comparisons were made by ANOVA for repeated measures, and Tukey's test as post-hoc and Students T test.

**Results:**

Long-term therapy decreased depression and binge eating symptoms, body image dissatisfaction, and improved QOL in girls, whereas, for boys, 24 weeks, were effective to reduce anxiety trait/state and symptoms of binge eating, and to improve means of dimensions of QOL (p < .05).

**Conclusion:**

A long-term multidisciplinary lifestyle therapy is effective to control psychological aspects and to improve QOL in obese adolescents.

## Background

Obesity has become an important public health problem worldwide, affecting different population groups on a pandemic scale [1,2]. Obesity is a chronic multifactor disease, that leads to multiple medical complications and psychological disorders [3,4]. In Brazil, recent data have shown that the prevalence of overweight and obesity in adolescent boys and girls was 16.1% and 17.5%, respectively [5].

Obesity is associated with physical problems, such as hypertension, coronary arteriosclerosis, elevated cholesterol, type 2 diabetes, joint problems, stroke, and certain types of cancers [6]. Psychologically, it is associated with several problems, such as lower self-concept, negative self-evaluation, decreased self-image, anxiety and depression [7], which are related to somatic and psychological symptoms (e.g. being teased, hit, or bullied, and presenting high-risk behavior) [8,9].

In addition, obesity has also adverse physical, social, and economic consequences that can negatively affect quality of life (QOL). As a result, QOL has become an important endpoint assessed in obesity and weight loss intervention studies [10].

However, few studies have examined the effects of lifestyle modification programs on changes in QOL among overweight and obese individuals. These studies suggest that physical activity combined with diet can be effective in improving health related QOL in several domains, including social functioning, mood, and self-esteem [11,12]. In general, obesity seems to have a greater impact on physical rather than mental functioning [13].

Therefore, current studies have shown that short-term weight loss has a positive effect on QOL in adults [7]. Nevertheless the effects of long-term therapies in adolescents have not been investigated. Thus the aim of this study was to verify the effects of a long-term multidisciplinary lifestyle intervention on QOL, body image, anxiety, depression and binge eating in obese adolescents.

## Methods

### Population

Obese adolescents were recruited from the Multidisciplinary Obesity Intervention Program outpatient clinic of the Federal University of São Paulo, in São Paulo, an urban city in Brazil. Sixty-six obese adolescents (41 girls and 25 boys; BMI: 35.62 ± 4.18 kg/m^2^) aged from 13–19 years old were included in the study. The inclusion criteria were: Tanner pubertal stage 3 or 4 (post pubertal) [14], primary obesity (BMI >95th percentile of the Centers for Disease Control reference charts) [15], and agreement of the adolescents and their families to participate, in a long-term multidisciplinary lifestyle therapy (24 weeks). The exclusion criteria were: identified genetic, metabolic or endocrine disease, chronic alcohol consumption, previous drugs use, and less than 75% compliance in all exercise, nutritional, psychological, and clinical sessions. Telephone surveys were conducted to investigate the reasons for dropping out of the program.

This study was carried out in accordance with the principles of the Declaration of Helsinki and was formally approved by the Ethics Committee of the Federal University of São Paulo – Paulista School of Medicine (#0135/04). Informed consent was obtained from all subjects and/or their parents.

### Study Protocol

**Figure 1:**
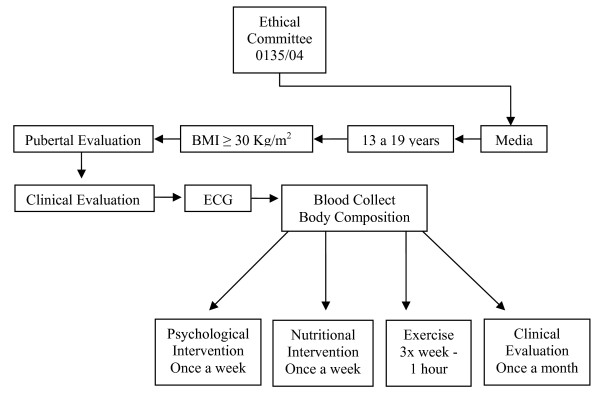
**Study protocol diagram**.

On the first visit, subjects were medically screened, had their pubertal stage assessed, and their anthropometrics profile measured (height, weight, BMI, and body composition) (Figure 1). For each subject, the procedures were performed at the same time of day and at least 15 hours after the last training session in order to avoid diurnal variations. Thereafter, obese adolescents started the multidisciplinary lifestyle therapy (short-term = 12 weeks and long-term = 24 weeks), composed of medical, dietary, exercise and psychological programs, as described bellow.

### Clinical Therapy

To accomplish healthy clinical parameters, the patients visited the endocrinologist once a month. Medical follow-up included initial medical history, physical examination, and appropriate tests followed by regular clinical surveillance.

### Psychotherapy

The adolescents participated in weekly psychological group sessions to discuss body image and eating disorders (bulimia and anorexia nervosa, binge eating, their signals, symptoms and their consequences for health), the relation between food and feelings, family problems among others. Individual psychological therapy was performed when nutritional or behavioral problems were identified.

### Nutritional Therapy

Nutritional lessons on food pyramid, recorded inquiry, weight loss diets, diet vs. light, fat and cholesterol, and nutrition facts were held once a week. No fixed caloric intake was prescribed; the subjects were only encouraged to reduce their food intake and follow a balanced diet.

### Physical Therapy

The adolescents underwent a personalized, moderate aerobic training program (60 min/session), three times a week (180 min/week), under the supervision of a sports physiologist. Each program was developed according to the results of an initial oxygen uptake test for aerobic exercises (cycle ergometer and treadmill). The exercise program was based on that of the American College of Sports Medicine [16]. Information about lifestyle changes related to activity was also provided and spontaneous physical activity (walking, stair climbing, etc.) was encouraged, but not measured.

### Anthropometric measurements and Body Composition

Subjects were weighed on a Filizola scale to the nearest 0.1 Kg wearing light clothing and no shoes. Height was measured to the nearest 0.5 cm by means of a wall-mounted stadiometer (Sanny, model ES 2030). BMI was calculated as body weight (kg) divided by height (m) squared (kg/m^2^). Body composition was estimated by Plethismography in the BOD POD^® ^body composition system (version 1.69, Life Measurement Instruments, Concord, CA) [17].

### Questionnaires

The following questionnaires were applied at baseline, after the short and long-term therapy:

1) STAI – Spielberger State-Trait Anxiety Inventory – a self-rated questionnaire divided in two parts: anxiety-trait (referring to personality traits) and anxiety-state (referring to systemic aspects of the context), translated into Portuguese and validated for the Brazilian population. Each part has 20 statements. Responses are in a 1–4 scale. Anxiety-state refers to how individuals feel 'at the moment', and anxiety-trait to how they 'generally feel'. Each part varies from 20 to 80 points, and the scores indicate low (0–30), medium (31–49) or high (50 or more) anxiety levels [18].

2) SF-36 – Health Research – Generic Questionnaire for Evaluation of Quality of Life 'Medical Outcomes Study SF-36' – Translated into Portuguese and validated for the Brazilian population. This is a multidimensional instrument consisting of 36 items to generically evaluate the QOL scored on 8 multi-item scales: physical functioning, role-physical, pain, general health perception, vitality, social functioning, role-emotional, mental health, plus a one-item measure of self-evaluated change in health status in the past year [19].

3) BSQ- Body Shape Questionnaire – Translated into Portuguese and validated for the Brazilian population. A 34-item measure of body dissatisfaction assesses the frequency of concern and distress about body size/shape. The subjects rate items on a scale from 1 (never) to 6 (always); higher scores reflect greater body dissatisfaction. The subjects were classified into light (between 81 and 110 points), moderate (between 111 and 140), and severe (more than 140 points) body image dissatisfaction [20].

4) BES- Binge Eating Scale – Translated into Portuguese and validated for the Brazilian population [21]. BES is a 16-item self-reported questionnaire, designed specifically to identify behavioral and cognitive characteristics of binge eating in obese individuals. Each item presents three or four differently weighted statements, with a final score varying from 0–46. It is used to identify binge eaters, to evaluate binge eating severity and also as a parameter for treatment outcome. Based on BES scores, disturbed eating behavior is classified into three different levels of severity: non-bingers (scoring 17 and less), moderate bingers (scoring between 18 and 26), and severe bingers eaters (scoring 27 and above) [22].

5) BDI – Beck Depression Inventory. The Brazilian version was used [23]. There are 21 clinical symptoms of depression, while the remaining 10 items cover affective, behavioral, somatic, and interpersonal aspects of depression. Each item consists of a series of four statements scaled to indicate increasing depressive symptomatology. Scores 5–9 indicate that these ups and downs are considered normal; 10–18, mild to moderate depression; 19–29, moderate to severe depression, and 30–63, severe depression. The scale was applied on the first day of the evaluation and after the training period [24].

The questionnaires were applied in a quiet room. Before beginning, the procedures were explained and the volunteers were asked to respond honestly.

### Statistical analysis

All data were analyzed by means of STATISTICA^® ^version 6 for Windows^®^, with significance set at p ≤ 0.05 and expressed as means ± SD.

Baseline, short and long-term multidisciplinary treatments were compared using analysis of variance (ANOVA) for repeated measures, and Tukey's post-hoc test was performed when necessary. Independent t-tests were used for comparison between genders. For comparisons between pre and post-treatment, effect size was expressed as a correlation and was displayed when it was up to moderate (>0.3) as proposed by Cohen [25].

## Results

In the present study, 63% (girls) and 77% (boys) completed 24 weeks of a multidisciplinary lifestyle therapy with more than 75% compliance in all exercise, nutritional, psychological, and clinical sessions. For both genders, the drop out rate before 12 weeks was 12%.

As expected, boys were heavier, taller and had more fat-free mass than girls in all evaluations. After the short-term multidisciplinary lifestyle therapy, both genders showed significant reductions in body mass, BMI and fat mass, without differences for 24 weeks, except a decrease in fat mass after the long-term therapy in girls. No changes in fat-free mass were observed after the treatment, and adolescents remained obese even with BMI reduction (32.04 ± 5.20 and 32.59 ± 4.50, for boys and girls respectively) (table 1).

**Table 1:** Body Composition and anthropometric profile of obese adolescents submitted to a multidisciplinary long-term lifestyle therapy.

Variable	Obese Girls			Obese Boys		
	
	Baseline	Short-term	Long-term	Baseline	Short-term	Long-term
Age (y)	16.56 ± 1.99	16.56 ± 1.99	16.56 ± 1.99	16.20 ± 2.09^c^	16.20 ± 2.09^c^	16.20 ± 2.09^c^
Height (m)	1.62 ± 0.05	1.62 ± 0.07	1.63 ± 0.10	1.72 ± 0.07^c^	1.72 ± 0.08^c^	1.74 ± 0.11^c^
Body Mass (kg)	94.45 ± 13.14	89.40 ± 13.01^a^	86.74 ± 12.73^a^	105.69 ± 9.72	89.40 ± 9.72^ac^	86.74 ± 11.31^ac^
BMI (kg/m^2^)	35.52 ± 4.19	33.47 ± 4.05^a^	32.59 ± 4.50^a^	35.78 ± 4.25	33.21 ± 4.38^a^	32.04 ± 5.20^a^
FFM (kg)	51.71 ± 5.65	49.83 ± 5.57	51.67 ± 5.45	66.67 ± 8.69^c^	63.69 ± 4.92^c^	64.70 ± 6.71^c^
Fat mass (kg)	41.91 ± 9.41	38.58 ± 9.36	35.35 ± 9.32^ab^	38.50 ± 10.05	32.79 ± 9.33^ac^	27.86 ± 11.03^ac^

ANOVA for repeated measurements followed by Tukey post-hoc test, p ≤ 0.05. ^a ^vs baseline; ^b ^vs short- term; ^c ^vs girls. BMI- Body Mass Index; FFM: Free Fat Mass

No genders differences were observed at baseline for symptoms of depression, anxiety state, binge eating and neither on lifestyle dimensions. However, girls had higher scores of anxiety trait and body image dissatisfaction before the therapy and after the long-term therapy, while boys had higher scores of physical function and pain (p < .05). No therapy effects were found after the short-term therapy in girls. On the other hand, after 24 weeks there were a decrease in depression symptoms (effect size = .36), body image dissatisfaction, binge eating symptoms (effect size = .38) and improvement on general healthy perception dimension of QOL. For boys, the short-term therapy improved body image dissatisfaction, and dimensions of QOL, such as physical function, role physical and role emotional (p < .05). Likewise, the long term-treatment was effective to reduce anxiety traits (effect size = .31) and state scores, and symptoms of binge eating, related to QOL. Twenty-four weeks of therapy improved vitality, role emotional and mean of dimensions. Moreover, the long-term therapy increased pain in obese males (p < .05) (Table 2).

**Table 2:** Descriptive scores of depression, anxiety, binge eating, body image dissatisfaction, and quality of life in obese adolescents submitted to a multidisciplinary long-term lifestyle therapy.

Variable		Girls			Boys		
		
		Baseline	Short-term	Long-term	Baseline	Short-term	Long-term
BDI		16.80 ± 7.61	15.41 ± 8.39	11.63 ± 5.19^a^	15.16 ± 8.32	13.04 ± 6.21	12.95 ± 8.31
STAI State		42.07 ± 9.01	43.00 ± 9.98	38.91 ± 11.13	39.96 ± 8.87	39.08 ± 11.62	35.60 ± 9.02^a^
BSQ		116.46 ± 34.23	112.80 ± 29.90	104.79 ± 28.14^a^	92.24 ± 32.39^c^	79.60 ± 25.73^ac^	77.84 ± 31.23^ac^
BES		15.53 ± 7.39	13.00 ± 8.08	10.25 ± 5.27^a^	14.60 ± 9.16	10.34 ± 9.30	7.45 ± 6.82^a^
SF-36	Physical Functioning	79.87 ± 16.33	83.05 ± 17.24	86.87 ± 9.30^a^	79.60 ± 18.42	89.34 ± 15.17^a^	93.50 ± 11.59^ac^
	Role Physical	76.21 ± 28.47	70.83 ± 34.06	77.08 ± 32.90	67.00 ± 33.63	80.43 ± 21.26^a^	78.75 ± 30.64^a^
	Pain	64.75 ± 15.78	71.80 ± 21.35	69.00 ± 19.86	73.64 ± 22.16	75.52 ± 20.14	83.85 ± 15.79^ac^
	General Health Perception	58.90 ± 19.71	64.13 ± 17.90	70.20 ± 17.54^a^	65.04 ± 16.14	71.95 ± 14.24	74.80 ± 21.72
	Vitality	61.82 ± 17.49	64.72 ± 18.93	70.62 ± 14.01	67.20 ± 19.20	70.86 ± 16.83	77.25 ± 17.50^a^
	Social Functioning	78.65 ± 19.81	78.81 ± 20.22	76.56 ± 24.53	79.00 ± 21.56	76.36 ± 20.74	80.62 ± 22.75
	Role Emotional	75.60 ± 30.75	67.58 ± 31.35	76.38 ± 33.30	62.66 ± 44.43	76.80 ± 21.16^a^	74.37 ± 35.31^a^
	Mental Health	69.75 ± 19.35	68.00 ± 21.42	74.66 ± 17.48	78.24 ± 13.27	77.91 ± 16.39	80.93 ± 16.51
	Mean of dimensions	70.70 ± 12.06	71.11 ± 15.41	75.00 ± 15.67	71.54 ± 17.20	77.43 ± 10.55	80.40 ± 14.09^a^

ANOVA for repeated measurements followed by Tukey post-hoc test, p ≤ 0.05. ^a ^vs baseline; ^b ^vs short-term; ^c ^vs girls. Legend: BDI = Beck Depression Inventory; STAI (Spielberger State-Trait Anxiety Inventory); BSQ = Body Shape Questionnaire; BES = Binge Eating Scale; SF-36 – Generic Questionnaire for Evaluation of Quality of Life;

## Discussion

Adolescent obesity is a multifactorial condition influenced by many factors. Previous studies have demonstrated that this type of multidisciplinary lifestyle therapy is effective to treat physical problems such as non-alcoholic fat liver disease [26,27], metabolic syndrome [28] and eating disorders [29], while others have shown that this kind of approach could be considered more effective than regular treatments that involve interventions in health alone [30,31].

Thus, we sought to examine several variables associated with psychological aspects. The main findings of this study were significant improvement in scores of depression, anxiety, binge eating, body image dissatisfaction, as well as domains of QOL. This is particularly important once several studies indicate that obese adolescents have a higher incidence of mental health problems, such as depression, anxiety, poor self-esteem and low QOL than non-obese adolescents, suggesting that this condition has a global impact on their daily life [32-35].

There is solid evidence in the literature supporting the assumption that cognitive behavioral interventions with adolescents are effective in decreasing depression and anxiety symptoms [36]. It is essential to understand the relationship between depression and obesity during adolescence, when both conditions may have their origins. Theoretically, depressed individuals eat to provide comfort or distraction from negative emotions [37]. Boys and girls have different patterns of depressive symptoms during puberty. Although they have an increase in depressive symptoms during adolescence, these symptoms are more dramatic in girls [33,38].

The significantly lower scores observed for depression and anxiety after short and long-term multidisciplinary lifestyle treatment and, consequently, the improvement observed in scores of mean dimensions of QOL can be explained by numerous factors, including increased self-esteem, stronger beliefs about the ability to engage in a healthy lifestyle related to healthier living attitudes, choices and behaviors [3].

A study comparing obese and non obese boys indicated that 44% of obese boys were not satisfied with their weight and 21% with their appearance. Therefore, obese boys reported more somatic and psychological symptoms, poor self-esteem and less healthy lifestyle. They feel unsuccessful and unhappy, and use drugs as a temporary medication [8]. As proposed in this study, we demonstrated a significant improvement of adverse effects on self-esteem and body image satisfaction in both genders. Nevertheless, body image dissatisfaction in girls was higher than in boys. Our results were in agreement with other findings in the literature [39,40], suggesting that a multidisciplinary therapy including psychological approaches, which encourages patients to change the way they think about themselves and their bodies in a more positive and realistic way, may help them to achieve crucial lifestyle changes needed for a better QOL.

Previous studies showed a positive correlation between binge eating disorders and obesity [37,41-43]. The present study revealed a statistically significant decrease in binge eating scores both in girls and boys after a long-term multidisciplinary therapy. These results can be attributed, at least in part, to a decrease in anxiety scores, since an anxious individual is more likely to develop binge eating disorders [4].

An important aspect observed in the present research was the beneficial effects of lifestyle intervention on QOL. In fact, obese boys had a significant improvement in physical functioning, role physical after the short and the long-term therapy; role emotional dimension after the short-term therapy; and after the long-term therapy they also presented an improvement in vitality. In girls, the beneficial effects observed were significant improvements in physical functioning and in general health. Another study found that obese adolescents present poor QOL when compared with normal weight adolescents, as demonstrated in all functioning domains, suggesting that their daily life is globally affected by this condition [44,45].

Aerobic exercise programs are related to better QOL scores, but physical exercise alone is not enough to promote a complete improvement. For any successful treatment, it is necessary to consider the individual in his/her totality (psychological, physical, social and behavioral aspects), and this is only possible in a multidisciplinary life-style therapy [26-29,46].

In addition, a multidisciplinary intervention may enhance health, facilitate and promote social contact, and favorably affect QOL, thus leading to improved social life and interaction [47]. Furthermore, it motivates people to adopt better lifestyle habits and it is an alternative treatment for stress since it has favorable impacts on every aspect of life.

Is important to note that the adolescents remained obese (at a lower degree) even losing weight. Therefore, we can attribute the improvements here described not only for weight loss, but a positive effect of the long-term multidisciplinary lifestyle therapy (24 weeks). John et al (2006) [48] failed in found an association between obesity and psychological disorders These findings should motivate obese individuals to seek for lifestyle interventions to treat obesity, focus on improved self-esteem, healthier choices, attitudes and healthier lifestyle behaviors, which can, at least, induce a better QOL, especially for subjects who do not respond to weight loss.

The drop out rate observed in our sample is consistent with other pediatric weight management programs [49-53]. Once the program was costless, we did not evaluate the socioeconomic status at the beginning of therapy. However, we identified, by phone calls that the drop out rate had been caused mainly due to the high cost of transportation, as the program center was distant from some of the volunteers' house. The hours demanded to complete each multidisciplinary session, can also be seen as a limitation of our study.

Other relevant limitations of this study are the lack of a control group, the relative small sample size, the lack of randomization and the absence of a follow-up. However, the original objective of the present study was to verify the effects of a long-term lifestyle therapy in obese adolescents. Despite these possible limitations, the effect size of our study is in accordance to the expectations of this type of therapy.

## Conclusion

The long-term multidisciplinary lifestyle therapy was effective to decrease symptoms of depression, anxiety, binge eating and body image dissatisfaction, which are essential to improve QOL in obese adolescents. Further researches involving obese adolescent's parents are necessary to identify the possible relations between family history and the development of psychological aspects observed in this study.

## Abbreviations

QOF: Quality of life; BMI: Body mass index; ECG: Electrocardiogram; STAI: Spielberger State-Trait Anxiety Inventory; SF-36: Short-Form 36; BSQ: Body Shape Questionnaire; BES: Binge Eating Scale; BDI: Beck Depression Inventory.

## Competing interests

The authors declare that they have no competing interests.

## Authors' contributions

MCLP: design, data collection, interpretation of data and drafting the manuscript. HKMA; WLP, AP, DAC, LT, JC: data collection, analysis and interpretation of data. ST, MTM and ARD: Design and critically revising of the manuscript. All authors read and approved the final manuscript.
